# Structure-guided machine learning prediction of drug resistance mutations in Abelson 1 kinase

**DOI:** 10.1016/j.csbj.2021.09.016

**Published:** 2021-09-16

**Authors:** Yunzhuo Zhou, Stephanie Portelli, Megan Pat, Carlos H.M. Rodrigues, Thanh-Binh Nguyen, Douglas E.V. Pires, David B. Ascher

**Affiliations:** a Systems and Computational Biology, Bio21 Institute, University of Melbourne, Melbourne, Victoria, Australia; b Computational Biology and Clinical Informatics, Baker Heart and Diabetes Institute, Melbourne, Victoria, Australia; c School of Chemistry and Molecular Biosciences, The University of Queensland, Brisbane, Australia; d Baker Department of Cardiometabolic Health, Melbourne Medical School, University of Melbourne, Melbourne, Victoria, Australia; e School of Computing and Information Systems, University of Melbourne, Melbourne, Victoria, Australia; f Department of Biochemistry, University of Cambridge, 80 Tennis Ct Rd, Cambridge CB2 1GA, UK

**Keywords:** Abelson 1 kinase, Mutations, Drug resistance, Structure-guided machine learning, Graph-based signatures

## Abstract

Kinases play crucial roles in cellular signalling and biological processes with their dysregulation associated with diseases, including cancers. Kinase inhibitors, most notably those targeting ABeLson 1 (ABL1) kinase in chronic myeloid leukemia, have had a significant impact on cancer survival, yet emergence of resistance mutations can reduce their effectiveness, leading to therapeutic failure. Limited effort, however, has been devoted to developing tools to accurately identify ABL1 resistance mutations, as well as providing insights into their molecular mechanisms. Here we investigated the structural basis of ABL1 mutations modulating binding affinity of eight FDA-approved drugs. We found mutations impair affinity of type I and type II inhibitors differently and used this insight to developed a novel web-based diagnostic tool, SUSPECT-ABL, to pre-emptively predict resistance profiles and binding free-energy changes (ΔΔ*G*) of all possible ABL1 mutations against inhibitors with different binding modes. Resistance mutations in ABL1 were successfully identified, achieving a Matthew’s Correlation Coefficient of up to 0.73 and the resulting change in ligand binding affinity with a Pearson’s correlation of up to 0.77, with performances consistent across non-redundant blind tests. Through an *in silico* saturation mutagenesis, our tool has identified possibly emerging resistance mutations, which offers opportunities for *in vivo* experimental validation. We believe SUSPECT-ABL will be an important tool not just for improving precision medicine efforts, but for facilitating the development of next-generation inhibitors that are less prone to resistance. We have made our tool freely available at http://biosig.unimelb.edu.au/suspect_abl/.

## Graphical abstract

**Figure:**
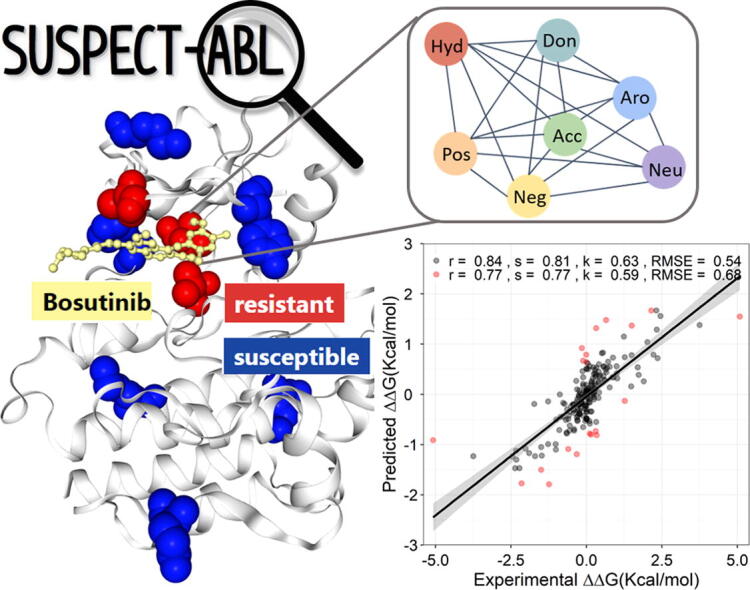

## 1 Introduction

Kinases can modulate protein activities through phosphorylation, acting as an essential on/off switch in many cellular signalling pathways. In chronic myelogenous leukemia (CML), ABeLson 1 (ABL1) kinase is constitutively overactivated as a consequence of chromosomal translocation with the breakpoint cluster region, and the induced abnormal cellular environment triggers malignant cell growth, initiating cancers [1]. The approval of imatinib in 2001, a first generation tyrosine kinase inhibitor (TKI), significantly improved cancer patient prognosis compared to the standard interferon alpha and cytarabine combination, highlighting the importance of targeting kinases in cancer treatment [2]. At the cellular level, imatinib competes with ATP to bind to, and consequently inhibit ABL1 kinase [3], [4], [5]. However, with increased clinical use over time, the therapeutic relevance of imatinib decreased due to the accumulation of missense mutations within its target, ABL1 kinase, especially those residues participating in drug recognition and binding. To overcome imatinib resistance, efforts were directed towards the development of second- and third-generation TKIs, such as dasatinib, nilotinib, bosutinib, ponatinib, and axitinib.

While several resistance mutation hotspots in ABL1 kinase (at gatekeeper, P-loop, αC-helix, and A-loop regions) modulating the binding affinity of different drugs have been well characterised [6], the increased mutation rate in cancers leads to the introduction of variants whose effects on therapeutic efficacy have not been previously studied. Pre-emptively identifying kinase mutations leading to potential therapeutic failure for specific drugs could lead to better and more personalised patient treatment and management.

One challenge for tackling ABL1 drug resistance is that mutations at the same position may modulate the drug affinity to a different extent, depending not only on the type of mutant amino acids but also on the drugs being used. Although most approved kinase inhibitors are ATP-competitive, they can be sensitive to different changes of the residue environment brought by different mutations, as each of them can have distinctive interactions with the kinase based on their favourable binding poses: (1) type I inhibitors bind to the active kinase (DFG-in), mainly occupying the hinge region where the adenine ring of the ATP binds; (2) type II inhibitors bind to the inactive kinase (DFG-out), extending to a hydrophobic back pocket while maintaining interactions with the ATP binding site. Apart from altering the drug affinity directly through the local atomic changes, mutations can also impact protein stability as well as dynamics, which may trigger conformational changes and impact drug recognition and interactions [7]. Moreover, these unknown structural changes may even cause favorable interactions with the endogenous ATP rather than the drugs, which could lead to the loss of drug competency and off-target effects. The broad repertoire of mutation effects and binding modes makes the characterization of molecular mechanisms of the resistance profiles a challenging task.

Experimental methods studying the effect of mutations on therapeutic efficacy, such as cell-based mutagenesis screening and yeast-based flow-cytometry [8], are labour intensive. Computational methods, on the other hand, have been proven useful and cost-effective for characterising the consequence of mutations. Although several predictive models have been developed to identify potential ABL drug resistance mutations [9], [10], [11], their performances were not sufficient to aid clinical decision support nor are they freely available for use or evaluation. Furthermore, limited insights into the molecular mechanism of drug resistance have been inferred from current models. Thus, there is a demand to develop computational tools capable of accurately predicting and understanding kinase drug resistance mutations.

We have previously shown that a suite of structural- and sequence-based computational tools which characterise the molecular consequence of mutations on protein dynamics, flexibility, stability, as well as ligand binding affinity, can successfully predict anti-tuberculosis drug resistance [12], [13], [14], [15], [16]. Additionally, we have also shown that the concept of graph-based signatures could be used to model both protein and small molecule structures, capturing both physicochemical and geometric properties [17], [18], [19]. Incorporating these two approaches, here we developed a novel, web-based diagnostic tool, SUSPECT-ABL (StrUctural Susceptibility PrEdiCTion for ABeLson 1 kinase), to pre-emptively predict the binding free-energy changes (ΔΔ*G*) and resistance profiles of all possible ABL mutations against eight FDA-approved drugs. The newly identified resistance mutations via *in silico* saturation mutagenesis offer opportunities for prioritization of *in vivo* experimental validation.

## 2 Results and discussion

**Fig. 1:**
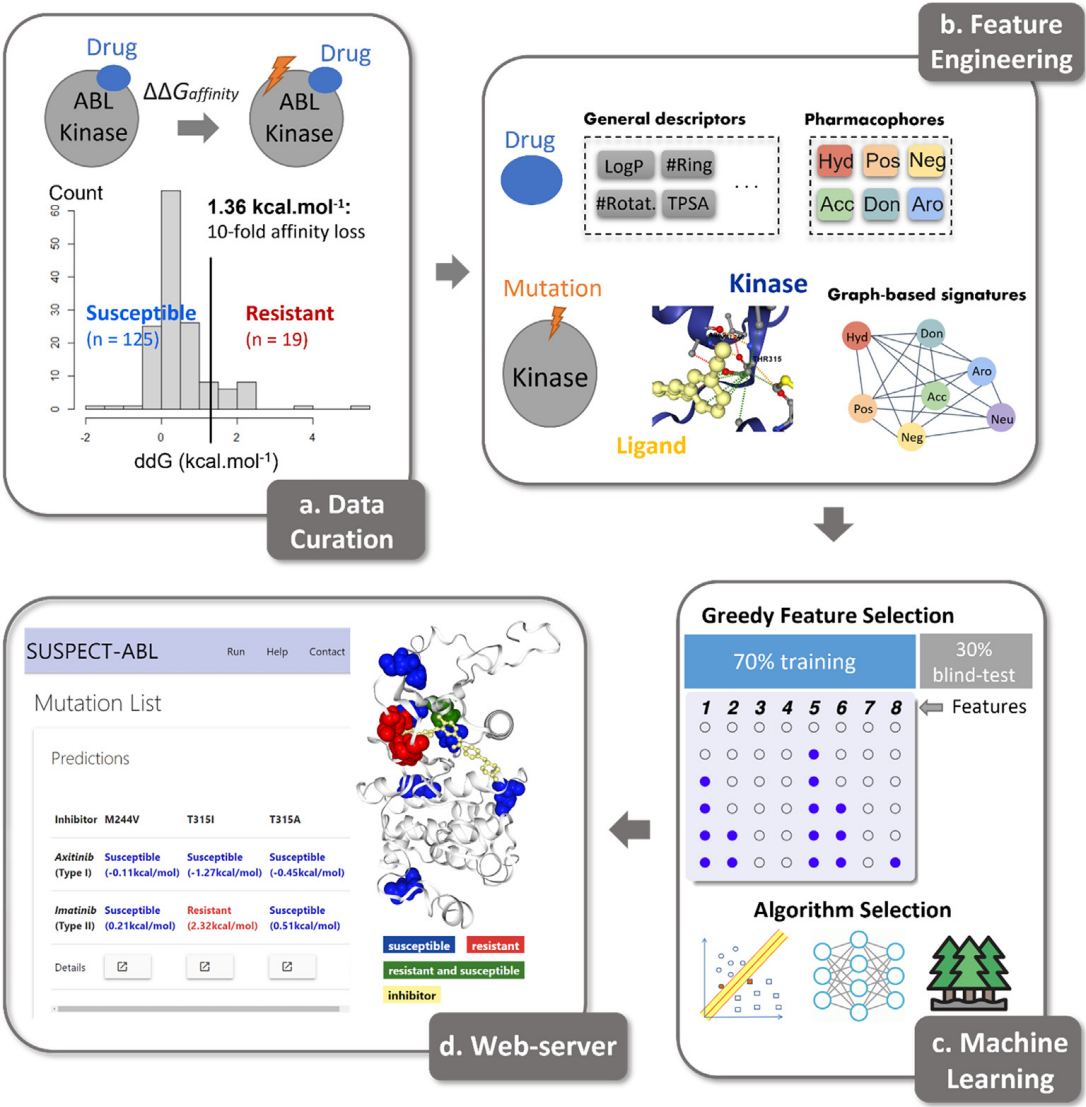
**Methodology workflow.** There were four steps involved in the methodology. Firstly, we curated the changes in binding free-energy upon mutation from Hauser *et al.*[9] and collected complex structures from RCSB PDB. After that, we generated a set of features capturing both physicochemical properties and graph-based patterns. These features were input into different machine learning algorithms, trained using cross-validation and tested on non-redundant blind test sets. Finally, a freely available web server, SUSPECT-ABL, was developed.

The methodology of this project is summarised in Fig. 1 by the following four steps: (1) data and structural curation, which involved molecular docking of inhibitors in the absence of experimental co-crystallized structures; (2) feature engineering, which involved the generation and evaluation of a set of features capturing the structural, geometric and physicochemical properties of the protein and ligands; (3) machine learning, where structural insights obtained were used to train and test supervised machine learning algorithms to accurately predict single-point mutations in ABL leading to resistance against eight inhibitors and the corresponding changes in Gibbs free energy of binding (ΔΔG); (4) web server development, where computational saturation mutagenesis results for eight FDA-approved drugs and the 3-dimensional visualization of ABL-ligand interactions were made freely available through a web server.

### 2.1 Structural information on ABL-ligand complexes

**Fig. 2:**
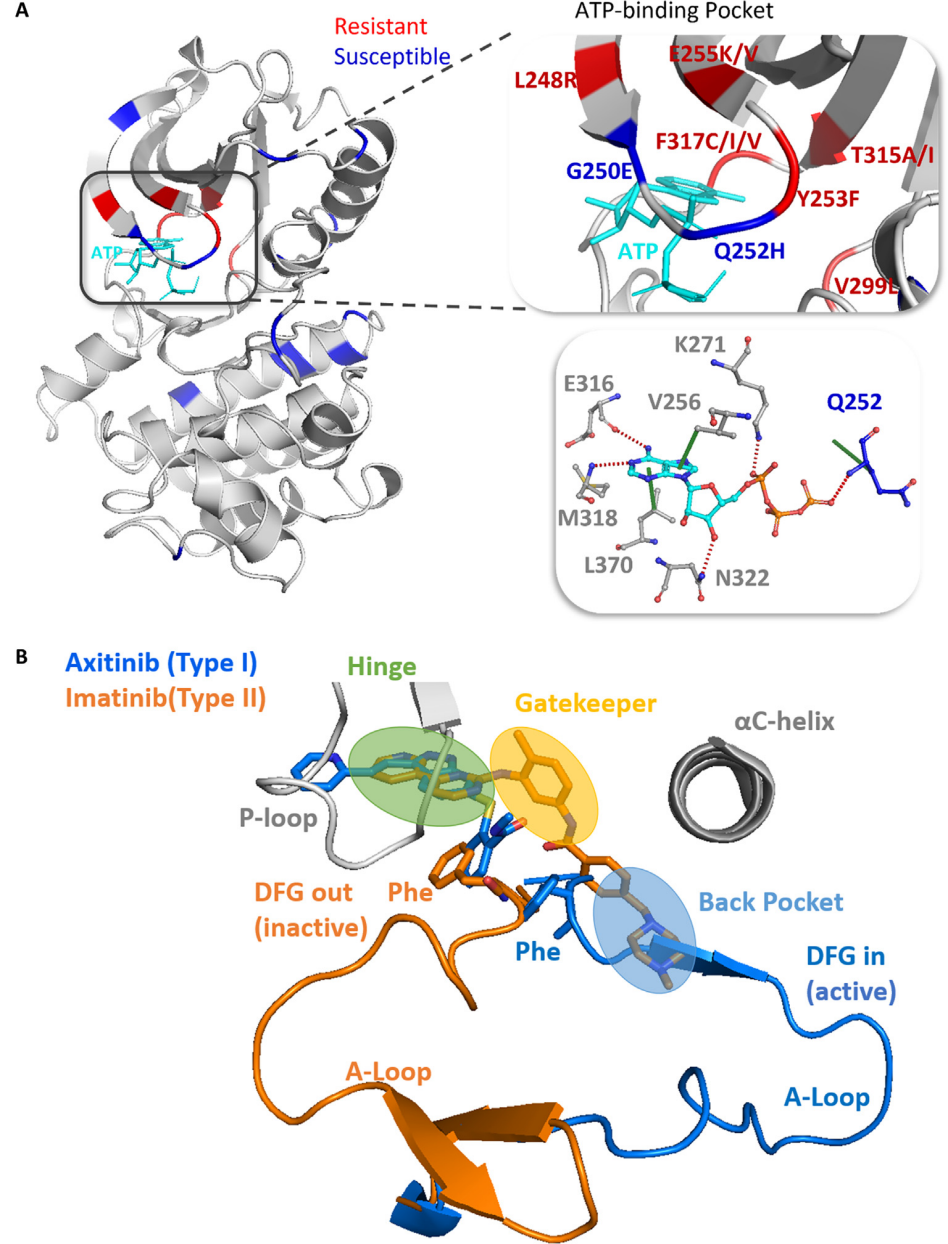
**Locations of mutations, inhibitors and the activation loop in ABL kinase. A)** Spatial distribution of mutations in eight ABL-drug complexes. The shown structure is ABL kinase complexed with ATP (PDB ID: 2G1T). Mutations causing resistance (ΔΔ*G* ≥ 1.36 Kcal/mol) to at least one drug are colored in red, other mutations (ΔΔ*G* <1.36 Kcal/mol) are colored in blue, and ATP interacting residues are shown in the bottom right box. **B)** The conformations of type I and type II binding modes. Type I inhibitors (*e.g.*, axitinib, in blue) bind to the active kinase, where the activation loop (A-Loop) adopts the DFG-in conformation (PDB ID: 4WA9). They occupy only the hinge region. Type II inhibitors (*e.g.*, imatinib, in orange) bind to the inactive kinase, where the A-Loop adopts the DFG-out conformation (PDB ID: 1OPJ). They have extra access to the gatekeeper and back pocket. (For interpretation of the references to colour in this figure legend, the reader is referred to the web version of this article.)

We curated 144 binding affinity effects (given as ΔΔG values) for 31 ABL mutations against eight FDA-approved drugs [9]. The corresponding ABL-ligand co-crystallized structures were curated from the RCSB Protein Data Bank [20] as available or generated by virtual docking [21], [22] (Table S1). The 31 mutations are located throughout the kinase domain, with some present at the ATP binding site, and multiple mutations clustering at the same position (Fig. 2A). Among them, six mutations were observed to cause resistance (as denoted by an experimental ΔΔ*G* ≥ 1.36 Kcal/mol, leading to more than 10-fold affinity loss) to at least one drug. Although these mutations were all located within the ATP binding pocket, we found the mutated residues are not essential for the binding of ATP, as shown by a lack of disruption to ATP-kinase interactions [23]. This may suggest that to cause drug resistance, the mutations should not impair the binding ability of ATP drastically.

Overall, the Abl-ligand complexes employed in this study are structurally diverse. This enables thorough analysis and predictions of resistance mutations throughout the kinase domain against different ligands with respective binding modes. The eight drugs include both type I (Axitinib, Bosutinib, Dasatinib, Erlotinib, and Gefitinib) and type II (Imatinib, Nilotinib, and Ponatinib) inhibitors. Superimposing the structures, we confirmed that the major conformational difference between the two binding modes is at the activation loop (Fig. 2B).

In the absence of Erlotinib- and Gefitinib-bound crystal structures, we docked these two inhibitors in the Bosutinib-bound structure (PDB ID: 3UE4) due to the shared type I binding mode and structural similarity among the three ligands. Similar to the quinoline group of Bosutinib, the recurring quinazoline group of Erlotinib and Gefitinib was thought to be the main determinant for binding, as it mimics the adenine ring of ATP. To confirm that the obtained poses of Erlotinib and Gefitinib were favourable, we calculated the intermolecular interactions using Arpeggio [23]. We found the quinazoline groups form hydrogen bonds with the hinge residue Met318, and Carbon-π interactions with Ala269 and Leu370, which is a shared characteristic with Bosutinib (Fig. S1). Moreover, the benzene rings connected to the quinazolines form Carbon-π interactions with the ABL1, which coincides with their interactions with epidermal growth factor receptor (EGFR) – the protein for which the Erlotinib and Gefitinib were originally developed as type I inhibitors.

### 2.2 Structural, biophysical, and evolutionary consequences of ABL mutations against different inhibitors

Although all resistance mutations in the dataset are located near the ATP site, the same mutation can modulate the binding affinity of type I and II inhibitors to a different extent due to their distinctive binding modes. We found some significant differences between resistant (ΔΔ*G* ≥ 1.36 Kcal/mol, n = 19) and susceptible (ΔΔ*G* <1.36 Kcal/mol, n = 125) mutations specific to each type of inhibitor.

**Fig. 3:**
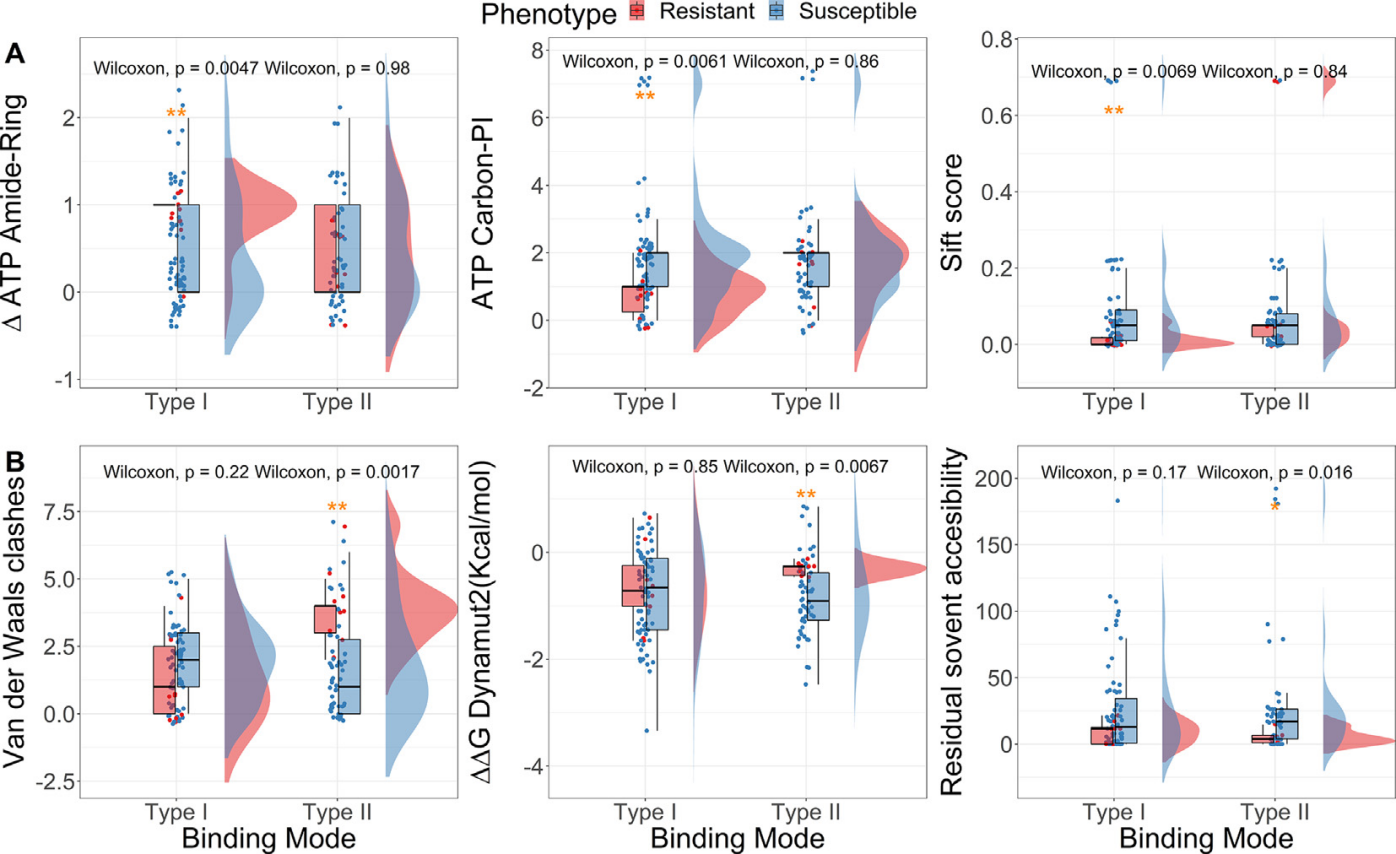
**Key features distinguishing resistant and susceptible mutations against type I (panel A) and II (panel B) inhibitors.** The structural, biophysical, and evolutionary consequences of resistance mutations are specific for each of the binding modes (* p-value < 0.05, ** p-value < 0.01, ‘Mann-Whitney’ test).

To better capture the cellular environments, we calculated the effects of mutations on both inhibitors and the endogenous ligand (ATP). Interestingly, resistance mutations against type I inhibitors were specifically found to be more associated with ATP modulation due to similar binding sites, even though the mutated residues do not directly interact with ATP. These mutations are more likely to impair an Amide-Ring interaction between residues, but fewer Carbon-π interactions were observed between the wild-type residues and ATP. Moreover, they have lower SIFT scores [24], which means the amino acid substitutions are more intolerant to the accumulation of mutations, resulting in detrimental effects on protein function (Fig. 3A, ‘Mann-Whitney’ test p-values <0.005). These findings suggest that resistance mutations against type I inhibitors may occur within the hinge region where the sequence is evolutionary conserved, but they may reduce the affinity of drugs more than the ATP. On the other hand, the affinity of type II inhibitors has been observed to be less sensitive to these mutations, as they extend to the extra pockets and reduce contacts with the hinge region (Fig. S2).

Resistance mutations against type II inhibitors, on the other hand, occur frequently in the phosphate-binding loop (P-loop). They usually involve more van der Waals clashes in the wild-type environment, destabilize the kinase to a lower extent, and have lower residual solvent accessibility (Fig. 3B, ‘Mann-Whitney’ test p-values <0.05). This can be caused by impairing the distinctive “kinked” conformation of the P-loop in the inactive ABL (Fig. S2), which is maintained by van der Waals clashes between several essential residues, and therefore decreasing the surface complementarity with type II inhibitors, favoring the ATP binding [25].

While drug binding affinity can be modulated by different structural, biophysical and evolutionary changes depending on the binding modes, we found that the gatekeeper mutation T315I can cause resistance to both types I and II inhibitors, with the exception of Axitinib (type I) and Ponatinib (type II). It has been shown that T315I causes resistance subject to a steric hindrance by a substantial conformational change from the inactive ABL (DFG-out) to the active form (DFG-in) [26], [27]. However, axitinib can still inhibit the T315I DFG-in mutant structure potently due to its distinct binding pattern – without close contacts with the activation loop [28]; and Ponatinib can effectively inhibit the conformational change of the activation loop, locking the T315I mutant complex at the DFG-out status without affecting ligand interactions [29], [30]. The overall higher efficacy of these two inhibitors across all the mutations can be associated with a common characteristic – having the lowest topological polar surface area (TPSA) within the respective binding modes, which makes them less sensitive to polarity change brought by mutations. Therefore, to better capture the impact of mutations on drug binding affinity, it is important to describe the residue environment using both structural and ligand information.

### 2.3 Quantifying the changes in binding affinity upon single point ABL mutations

Based on the patterns observed through structural analysis of the ABL-ligand complexes and the molecular properties of ligands, we next sought to quantitatively predict the changes in ΔΔ*G* upon missense mutation in response to each of the drugs using supervised machine learning. Different machine learning algorithms from python sklearn library [31] were used to train and optimize regression models through bottom-up greedy feature selection on 204 ΔΔ*G* values, which included 102 ΔΔGs obtained from hypothetical reverse mutations, generated to better balance the distribution of values, which are naturally biased towards mutations decreasing binding affinity. Of these, the Extra Tree regressor was chosen based on better and consistent performance between cross-validation and external blind test set, measured through Pearson’s correlation. The final model, SUSPECT-ABL was made up of 10 features, which broadly describe the protein local environmental changes upon mutation, ligand properties and sequence-derived evolutionary properties (Table S3).

**Fig. 4:**
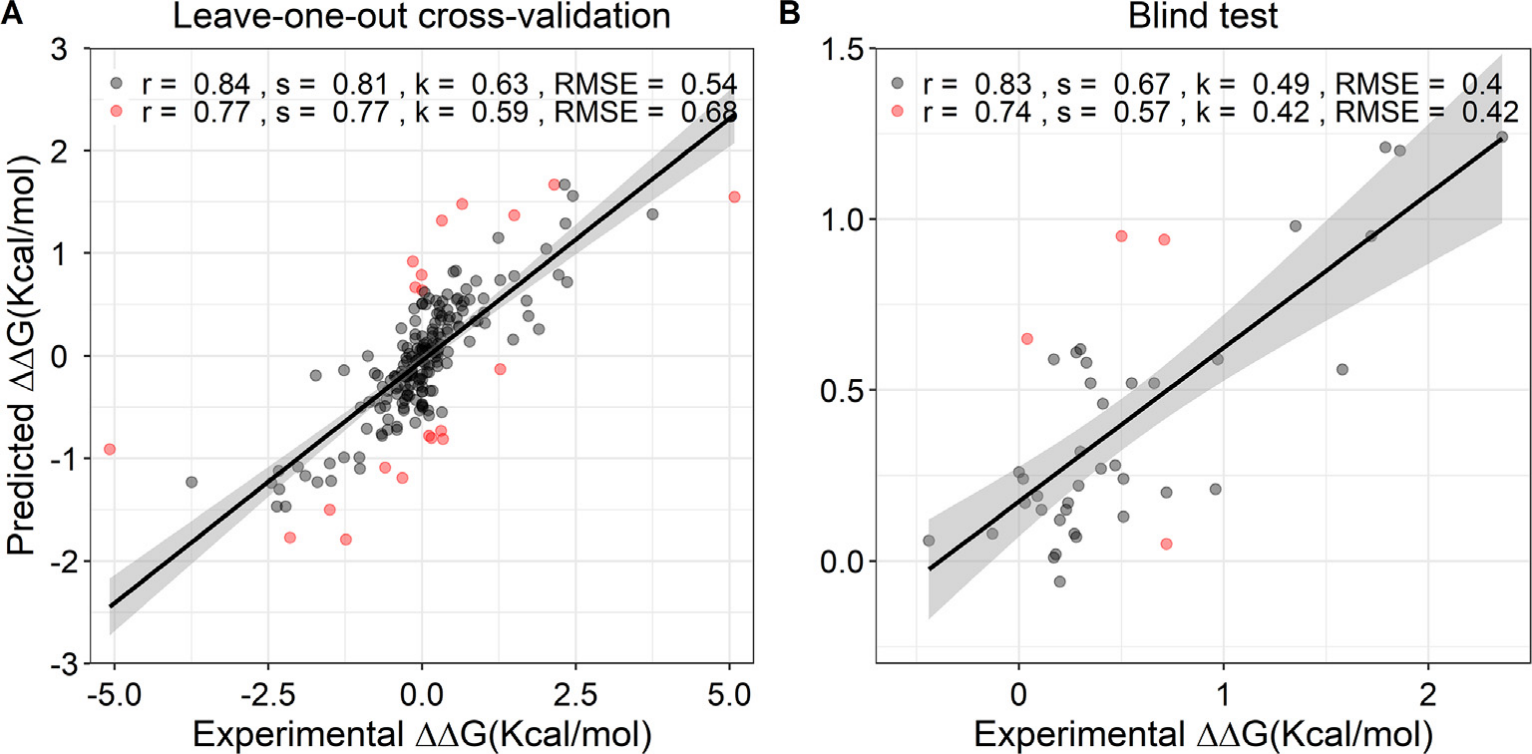
**Regression plot between actual and predicted ΔΔG on low-redundancy cross-validation (leave-one-position out) (102 forward and 102 hypothetical reverse mutations, left panel) and on the independent blind test set (42 forward mutations, right panel).** By removing the 10% outliers (highlighted in red), the performances improved. (For interpretation of the references to colour in this figure legend, the reader is referred to the web version of this article.)

On a low-redundancy validation scheme, SUSPECT-ABL was able to achieve Pearson's, Spearman's and Kendall's correlations of 0.77, 0.77 and 0.59 respectively (RMSE = 0.68 Kcal/mol) on the training set, under leave-one-position-out cross-validation; and 0.74, 0.57 and 0.42 (RMSE = 0.42 Kcal/mol) on the blind test (Fig. 4), significantly outperforming other models which use data-driven or physics-based computational approaches (Table 1). After removing the 10% outliers, Pearson’s correlations increased to 0.84 on cross-validation and 0.83 on the blind test. These outlier mutations were all located in beta strands. Among them, the hypothetical reverse mutations lay mostly below the best-fit line (underestimated), whereas the predictions for their respective original forward mutations lay above (overestimated). This can be caused by compromising several strong resistance mutations in beta strands. Unlikely, the 10% outliers in the blind test set are all located at position 317 in a loop, which is thought to be a result of its overrepresentation (64%) in the blind test set. The performance when including only the forward mutations, and using 10-fold cross-validation are shown in Table S2 for comparison purposes.

**Table 1:** **Performance of the final regressor on a non-redundant blind test set.** The model was trained including both forward and hypothetical reverse mutations, and tested on a non-redundant blind test set. The performances of other methods are also shown for comparison purposes.

Name	Method	Pearson	Spearman	Kendall	RMSE (Kcal/mol)
**SUSPECT-ABL**	**Machine Learning**	**0.74**	**0.57**	**0.42**	**0.40**
mCSM-lig [49]	Machine Learning	0.43	0.33	0.24	0.75
Aldeghi *et al.*[11]	Machine Learning	0.06	0.21	0.15	0.64
**Hauser *et al.***[9]	**Molecular Dynamics**	**0.65**	**0.38**	**0.30**	**0.69**
A99 [11]	Molecular Dynamics	0.40	0.26	0.17	0.68
A99L	Molecular Dynamics	0.60	0.37	0.26	0.55
A99DC	Molecular Dynamics	0.58	0.45	0.31	0.55
A14	Molecular Dynamics	0.37	0.37	0.26	0.70
**R15**[11]	**Rosetta**	**0.72**	**0.50**	**0.38**	**0.49**
R16	Rosetta	0.47	0.29	0.20	0.70

Among the 10 selected features (Table S3), 7 are graph-based signatures that capture the geometric properties of atoms within the wild-type local residue environment, describing the 3-dimensional inter- and intra-atomic arrangements in both ABL-drug complexes and the ABL-ATP complex. Additionally, there are two ligand features, positively ionizable atom counts and the number of rotatable bonds, which potentially distinguish type I and II inhibitors as well as the higher effectiveness of axitinib and ponatinib in general. Lastly, a sequence-based feature that relies on the optimal substitution matrix, incorporating both multiple sequence alignment and the evolutionary information in the context of secondary structure, KOSJ950100_SST (aaindex[32], [33]), could potentially capture the impact of mutations on protein structure.

Based on such diverse features ranging from the geometric properties of ABL-ligand structures, ligand properties, and the structural impact of mutations, we further investigated whether SUSPECT-ABL shows similar performance on each of the binding modes. By expanding the prediction results, we found that although the Pearson’s correlations are inconsistent across the two binding modes on training and blind test sets, this could be caused by limited sample size and high similarity between samples in the blind test set (Fig. S3). On the other hand, the model achieved reasonable RMSE for both types I (0.74 Kcal/mol on cross-validation, 0.46 Kcal/mol on blind test) and II (0.61 Kcal/mol on cross-validation, 0.34 Kcal/mol on blind test) inhibitors. Therefore, we still expect that SUSPECT-ABL could generalize well on novel data for both binding modes.

### 2.4 Classifying resistant and susceptible ABL mutations

**Fig. 5:**
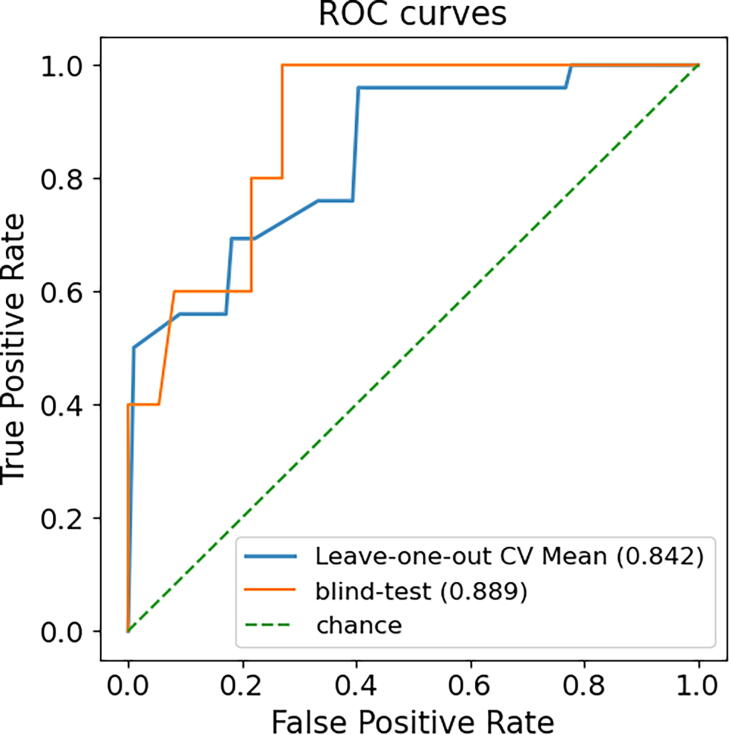
**ROC curves comparing the classification model performance on the cross-validation and the blind test set.** Our model was able to correctly identify resistance mutations with AUC >0.8 for both training and blind test sets.

While the binding free-energy changes can give us insights into how strongly the mutations affect drug affinity, for clinical applications, it can also be valuable to directly predict whether a given mutation could lead to drug resistance. Rather than transforming the predictions from our regressor to resistant (ΔΔ*G* ≥ 1.36 Kcal/mol) and susceptible (ΔΔ*G* <1.36 Kcal/mol) mutations using the defined 10-fold affinity loss threshold, we decided to train a separate classifier to improve the model robustness. To keep consistency, the dataset was split into training and blind test in the same way. Since only a few mutations increase the drug binding affinity (ΔΔ*G* <0), the inclusion of hypothetical reverse mutations does not mitigate the data imbalance (19 resistant and 125 susceptible mutations). With such limited samples of resistance mutations, however, SUSPECT-ABL still achieved Matthew’s correlation coefficient (MCC) of 0.73 (AUC = 0.84, Fig. 5) on the training set under stratified leave-one-position-out cross-validation, and 0.63 (AUC = 0.89) on the blind test, using the Random Forest classification algorithm. The performance when including the hypothetical reverse mutations, and using 10-fold cross-validation are shown in Table S4 for comparison. Notably, the nine selected features in the classifier overlap significantly with the key features distinguishing resistant and susceptible mutations detected previously in Fig. 3 (TPSA, Δ Amide-Ring interaction, and DynaMut2[34], [35]), which further highlights their importance (Table S5).

Compared to several other models, the state-of-the-art molecular dynamics method by Hauser *et al.* [9] and the Rosetta method R15 achieved higher MCCs on the blind test set (0.77 and 0.76, respectively), but their significantly lower MCCs on our training set (0.30 and 0.43, respectively) indicates that these two methods can be highly sensitive to different mutations (Table 2). By analysing the false negatives (resistance mutations which are predicted as susceptible), we found SUSPECT-ABL, Hauser *et al.* [9] and R15 were not able to identify the mutation E255V, which is resistant to imatinib and nilotinib, as well as the mutation E255K being resistant to only imatinib.

**Table 2:** **Performance of the final classifier on a non-redundant blind test set.** The model was trained using leave-one-position out cross-validation methods including only the forward mutations, and tested on a non-redundant blind test set. For the purpose of this classification, resistance mutations were those having a binding affinity higher than 1.36 kcal/mol. The performances of other methods are also shown for comparison purposes. Namely, methods adopted by Hauser *et al.* and Rosetta outperformed SUSPECT-ABL, despite poor performance on our training set. The F1 score was calculated when the resistant class is the positive, and the susceptible class is the negative.

Name	Method	MCC	F1	BACC
**SUSPECT-ABL**	**Machine Learning**	**0.63**	**0.67**	**0.79**
**Hauser *et al.***[9]	**Molecular Dynamics**	**0.77***(0.30 on our training set)*	**0.80**	**0.89**
A99/A99L/A99DC [11]	Molecular Dynamics	0.42	0.33	0.6
A14	Molecular Dynamics	−0.06	*NaN*	0.49
**R15**	**Rosetta**	**0.76***(0.43 on our training set)*	**0.77**	**0.96**
R16	Rosetta	0.55	0.60	0.77

The molecular interactions calculated by Arpeggio [23] revealed that E255 has no direct contact with the ligands, instead, its carboxylate group in the side chain forms an electrostatic triad with K247 and Y257, stabilizing the P-loop, which was proven to be required for binding of Type II inhibitors [25] (Fig. S4). Disruption of the triad interactions, however, can reduce the surface complementarity with drugs, and cause distributed allosteric effects which destabilize the activation loop. This rare and indirect contribution to drug resistance increases the challenge for detection. Interestingly, after we changed the resistance threshold from 10-fold affinity loss to 5-fold (ΔΔ*G* = 0.95 Kcal/mol), the model was able to detect five out of six resistance cases of E255 mutations, while still predicting the susceptible cases correctly. Moreover, the overall performance of the 5-fold affinity loss classifier (MCCs of 0.75 on training, 0.67 on blind test) improved compared to the 10-fold (Table S6), which can be caused by the increased sample size of resistance cases (from 19 to 27). Therefore, the indirect contributions of resistance can still be detected while relaxing the classification threshold.

### 2.5 Deploying the model for saturation mutagenesis

We performed *in silico* saturation mutagenesis within the kinase domain to pre-emptively identify ABL mutations leading to therapeutic failures. Although the resistance mutations (more than 10-fold affinity loss) in the dataset were clustered within the ATP binding pocket, several studies [25], [36] have shown that distant mutations may also impair drug binding, despite many of them occurring less frequently. To test whether our classification model could generalise to those unseen mutations, we thoroughly analysed the newly identified resistance mutations that occur at different positions with those in the dataset created by Hauser *et al.* [9], and found that many of them coincide with clinically observed or *in vitro* screened resistance mutations from various sources [25], [36]. These include mutations at residues close to the ATP site, which have no direct contact with ATP but shields the corresponding drugs from solvent (V268, A269, V270, and G321); as well as mutations at allosteric sites to ATP, which destabilize the ABL-drug complex by disrupting the gatekeeper residue or the activation loop (F283, M290, and F382). Thus the resistance mutations predicted by SUSPECT-ABL through saturation mutagenesis are not restricted to the residues within the ATP pocket which directly interact with the drugs, but extend to more complicated resistance mechanisms by including biophysical features in the model.

### 2.6 SUSPECT-ABL web server

We have implemented SUSPECT-ABL as a user-friendly and freely available web server at http://biosig.unimelb.edu.au/suspect_abl/, which is a database for all possible ABL variants within the kinase domain (from residue number 242 to 493). The web server allows the predictions of phenotypes (resistant or susceptible) and the changes in ΔΔG upon missense mutation, as well as the visualization of molecular interactions within the wild-type and mutant residue environment. Users can select one or more FDA-approved tyrosine kinase inhibitors, specify a single missense mutation in the format T315I (where threonine is the wild-type residue, 315 is the residue position, isoleucine is the mutant residue), or upload a list of missense mutations in the same format. A step-by-step help page with illustrative figures can be found at http://biosig.unimelb.edu.au/suspect_abl/help.

**Fig. 6:**
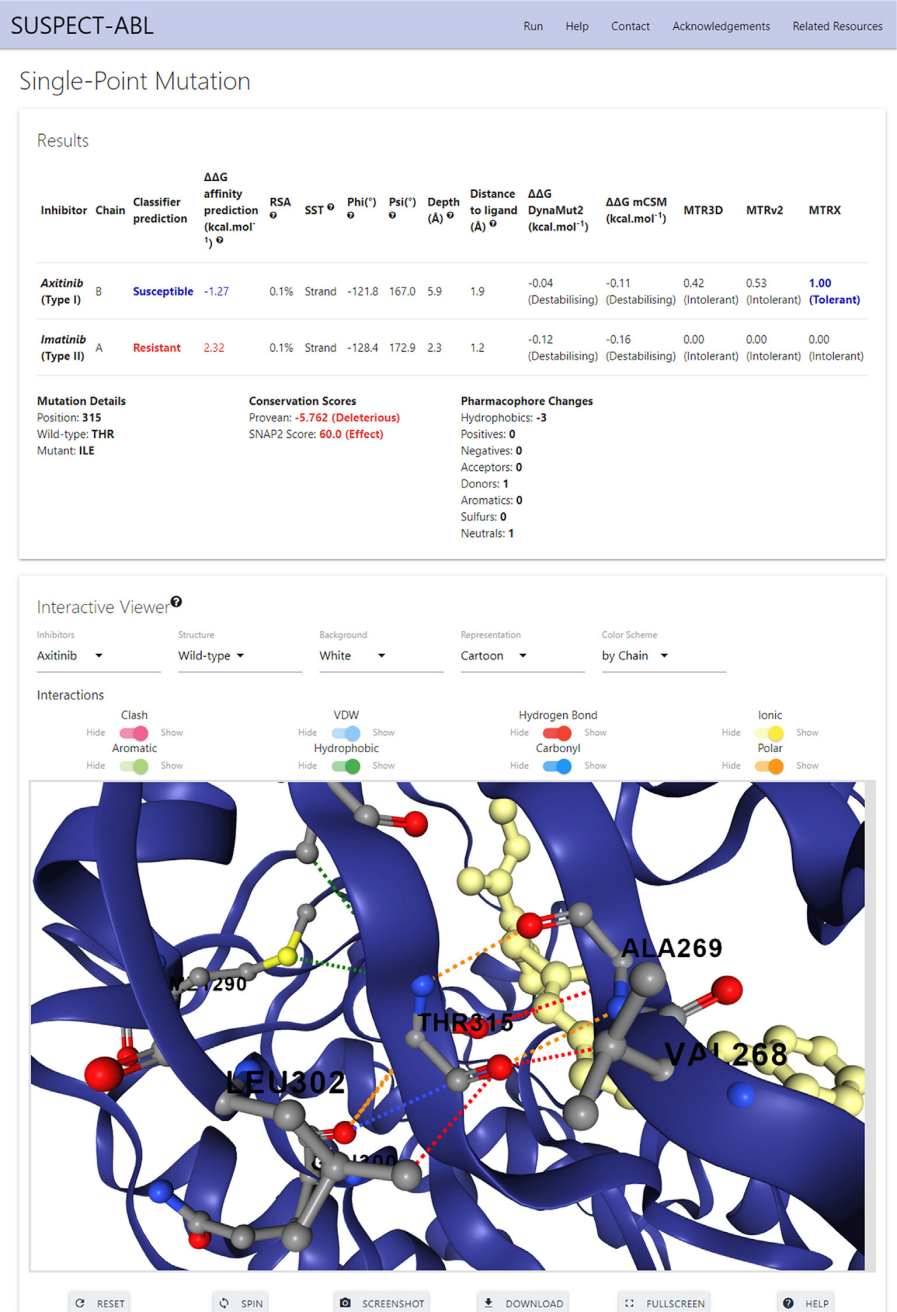
**SUSPECT-ABL web server interface for single point mutation input.** The predictions for the regressor and the classifier are displayed in a table, along with the information about the wild-type environment and several other predicted parameters. Mutation details, conservation score and pharmacophore changes are also shown below the table. In the interaction viewer box, users can visualise the non-covalent interactions around the mutation site, and are allowed to select different inhibitors, compare the wild-type and mutant, as well as customize the representation.

Fig. 6 shows a snapshot of the output page for the single mutation T315I while selecting axitinib and imatinib. The web server displays the results in a table, where rows are inhibitors, columns include the predictions of phenotypes, ΔΔ*Gs*, information about the wild-type environment (residual solvent accessibility, secondary structure, dihedral angles, residual depth [37], and distance to ligand) and several other predicted parameters (DynaMut2 [34], [35], mCSM [17], MTR3D [38], MTRv2 [39], [40], and MTRX scores). Below the table, there is information about the mutation, its conservation scores predicted by PROVEAN [41] and SNAP2 [42], as well as the resulting pharmacophore changes. Additionally, there is a downloadable 3D interactive viewer built using NGL [43], which allows users to analyse the non-covalent intermolecular interactions for the residue specified in the input calculated using Arpeggio [23], for both wild-type and mutant structures across different ABL-inhibitor complexes. The results for the mutation list input are summarized in a downloadable table, where rows are drugs and columns are mutations, which allows users to compare the predicted phenotypes and ΔΔ*G*s for different drugs across input mutations, and access the corresponding result pages for single mutations through links. There is also a 3D viewer at the bottom of the page in which the residues in the input mutation list are mapped to the ABL-ligand complexes, and colored according to the predicted phenotype (Fig. S5).

## 3 Conclusions

Here we present SUSPECT-ABL, a web server that integrates our well-established graph-based signatures concept with dynamics properties, conservation scores and small-molecule properties to accurately predict how missense mutations in ABL1 kinase modulate the binding affinity of eight FDA-approved drugs and thereby lead to resistance. Our method has shown to be robust when evaluated using different cross-validation methods, and outperformed the existing tools on a non-redundant blind test set. Since the ATP sites are highly conserved across different kinases, this approach can also be potentially applied to identify resistance mutations in other kinases against any given ATP-competitive kinase inhibitor, using either the experimental kinase-ligand structures or docked structures. We anticipate expanding this tool to predict the impact of kinome-wide missense mutations on modulating the inhibition ability of a large library of small molecule kinase inhibitors. Finally, due to its consideration of ABL1-inhibiting drugs with different clinical indications, our tool, SUSPECT-ABL offers opportunities to both clinical and research fields. This is particularly due to its applicability in drug repurposing, and hit prioritization according to resistance potential detectable through large-scale comparisons across mutations followed by *in vivo* experimental validation. In this way, our tool could reduce cost and time commitment in the pipeline of improving precision medicine, and eventually providing patients with tailored chemotherapies.

## 4 Methods

### 4.1 Dataset

The binding affinity effects of 144 mutations (ΔΔG, given in Kcal/mol*)* were obtained from Hauser *et al.* [9], including 19 resistant (ΔΔ*G* ≥ 1.36 Kcal/mol) and 125 susceptible (ΔΔ*G* <1.36 Kcal/mol) mutations defined by the 10-fold affinity loss cut-off. Within this dataset, mutations were phenotyped according to their response to eight different TKIs: imatinib, dasatinib, nilotinib, bosutinib, ponatinib, erlotinib, gefitinib and axitinib. Of these, all drugs have shown strong inhibition to ABL1 kinase *in vitro* [28], [44], while imatinib, dasatinib, nilotinib, bosutinib and ponatinib are indicated clinically for use in CML. Further to that, given the limited sample size of the original dataset, we introduced hypothetical reverse mutations in the training set as an over-sampling strategy to better balance the range of mutation effects and increase model robustness for the regression task. These reverse mutations were computed based on the premise that the binding free energy change of a mutation from the wild-type ABL to its mutant (ΔΔ*G*_WT→MT_) is equivalent to the opposite change in binding free energy of the hypothetical reverse mutation (-ΔΔ*G*_MT→WT_) [35], [45].

### 4.2 ABL-ligand structures

The six available ABL-ligand co-crystallized structures were curated from the RCSB PDB [20] (Table S1), and preprocessed in Maestro to fill in missing residues and atoms. Of these, the imatinib-bound crystallized structure (PDB: 1OPJ), represented the mouse homolog, and was used as a template for homology modelling of the human ABL1 kinase. Additionally, when processing the ATP-bound structure (PDB: 2G1T), we removed the peptide conjugate present with ATP, as this was required for crystallization, but not for ATP binding. Finally, as the crystallographic structures of ABL1 kinase bound to erlotinib and gefitinib were not available, these drugs were docked based on the bound coordinates of co-crystallized Bosutinib (PDB: 3UE4), due to similarities in chemical moieties as calculated using ChemMine Tools, and binding modalities (type I inhibitors). During docking, the methods EasyVS [22] and Glide from the Maestro package [21] were used for comparison purposes. An initial redocking of Bosutinib within its crystal structure was used as a control to assess the potential utility of each tool within the target ABL1 kinase, and yielded comparable poses to the co-crystallized ligand. Comparisons between all binding poses were carried out using Pymol, where the final chosen poses were generated using Maestro, and retained comparable contacts between the drug and receptor, calculated using Arpeggio.

### 4.3 Feature generation

The structural consequences of all ABL mutations were captured by considering a total of 1,013 features, which were broadly divided into descriptors of (1) the molecular properties and pharmacophore modelling of different drugs; (2) the physicochemical properties and local flexibility of ABL around the mutation sites; (3) the interatomic interactions between ligands and the kinase; and (4) the distance patterns between pharmacophore pairs within the local residue environment using the concept of graph-based signatures (Fig. 1B).

The first feature subset described the different ligands bound to ABL1. It includes molecular properties (logP, number of rings, rotatable bonds, surface area and others) calculated by the Python RDKit toolkit based on SMILES, and the atomic pharmacophore frequency counts [18], [19], [46], [47], [48]. The distances from ligands to the mutation sites in Angstrom (Å) and ΔΔG predicted by mCSM-lig [49] were also included.

The second feature subset considered the unbound ABL kinase, capturing the impacts of mutations on physicochemical properties at the binding site (aaindex [32] and changes in pharmacophore count[17]), protein stability changes to account for the local conformational and flexibility changes (predicted by Dynamut [34], Dynamut2 [35], mCSM [17], DUET [50], and SDM[45]), and functional changes predicted by SIFT [24]. Furthermore, other features considering the local residue environment in wild-type structures included residue depth [37], residue solvent accessibility, deformation and fluctuation energies [51].

The third feature subset included the local interatomic contacts of ligand-kinase complexes calculated by Arpeggio, both in the wild-type and the differences between the wild-type and the mutant. These features were calculated for each bound ligand, and compared to endogenous ligand ATP.

The final subset is the graph-based signatures [17], which capture the geometric properties within the local residue environment under different distance thresholds. The atoms with pharmacophore labels (Hydrophobic, Positively charged, Negatively charged, Hydrogen Acceptor, Hydrogen Donor, Aromatic and Neutral) are modelled as nodes, and atomic interactions are modelled as edges. From this graph representation, distance patterns between pharmacophore pairs around the mutation site are captured as a cumulative distribution.

### 4.4 Machine learning and performance evaluation

To ensure low-redundancy, the dataset was split in a way that mutations at the same site occur exclusively in the training (102 ΔΔ*G* values for 19 mutations across 13 sites) or in a non-redundant blind test set (42 ΔΔ*G* values for 12 mutations across 6 sites). We trained and evaluated the models using different controls and comparisons for both the regressor and the classifier: with and without reverse mutations; 10-fold versus leave-one-position-out cross-validations within the training set; as well as different machine learning algorithms from the python sklearn library [31] (random forest, extra trees, multilayer perceptrons, and support vector machines). The ones with the best performance were selected as our final models.

Moreover, a bottom-up greedy feature selection method was used to reduce the noise, according to Pearson's correlation (r) for the regressor, and Matthew’s correlation coefficient (MCC) for the classifiers. The final models were chosen based on consistent performance between train and test. The model performances were further evaluated by different metrics: Kendall's and Spearman's correlation coefficients, and root mean squared error (RMSE) for regression; F1 score, balanced accuracy (BACC) and Area Under receiver operating characteristic Curve (AUC) for classification.

### 4.5 Web server development

The SUSPECT-ABL server front-end was built using materialize CSS framework version 1.0.0, while the backend was built in Python via the Flask framework (version 0.12.2). It is hosted on a Linux server running Apache.

## Declaration of Competing Interest

The authors declare that they have no known competing financial interests or personal relationships that could have appeared to influence the work reported in this paper.
