# Supplementary material for: Structure-guided machine learning prediction of drug resistance mutations in Abelson 1 kinase
Source: Comput Struct Biotechnol J. 2021 Sep 16;19:5381–91. doi: 10.1016/j.csbj.2021.09.016 (PMC8495037; doi:10.1016/j.csbj.2021.09.016)
Supplement: Supplementary data 1 [file mmc1.docx]

**SUPPLEMENTARY MATERIALS**

Structure-guided machine learning prediction of drug resistance mutations in Abelson 1 kinase

Yunzhuo Zhou^1,2^, Stephanie Portelli^1,2^, Megan Pat^1,2^, Carlos H. M. Rodrigues^1,2,3^, Thanh-Binh Nguyen^1,2,3,4,*^ , Douglas E.V. Pires^1,2,3,5,*^, David B. Ascher^1,2,3,4,6,*^

^1^ Systems and Computational Biology, Bio21 Institute, University of Melbourne, Melbourne, Victoria, Australia
^2^ Computational Biology and Clinical Informatics, Baker Heart and Diabetes Institute, Melbourne, Victoria, Australia
^3^ School of Chemistry and Molecular Biosciences, The University of Queensland, Brisbane, Australia
^4^ Baker Department of Cardiometabolic Health, Melbourne Medical School, University of Melbourne, Melbourne, Victoria, Australia

^5^ School of Computing and Information Systems, University of Melbourne, Melbourne, Victoria, Australia
^6^ Department of Biochemistry, University of Cambridge, 80 Tennis Ct Rd, Cambridge CB2 1GA, UK

*To whom correspondence should be addressed D.B.A. Tel: +61 90354794; Email: [david.ascher@unimelb.edu.au](about:blank). Correspondence may also be addressed to T.N. [thanhbinh.nguyen@unimelb.edu.au](mailto:thanhbinh.nguyen@unimelb.edu.au) or D.E.V.P. at [douglas.pires@unimelb.edu.au](mailto:douglas.pires@unimelb.edu.au).

#

# **TABLES**

## Table S1. Structures and mutations of inhibitor-bound ABL kinases from Hauser *et al.*^1^

| **Inhibitors** | **PDB** | **Binding Mode** | **Resolution (Å)** | **No. of Resistant Mutations** | **No. of Susceptible Mutations** | **Δ*G*_WT_**  **(Kcal/mol)** |
| --- | --- | --- | --- | --- | --- | --- |
| Axitinib^2^ | 4WA9 | Type I | 2.20 | 0 | 26 | -8.35 |
| Bosutinib | 3UE4 | Type I | 2.42 | 4 | 17 | -9.81 |
| Dasatinib | 4XEY | Type I | 2.89 | 5 | 16 | -11.94 |
| Erlotinib | Dock to 3UE4 | Type I | - | 1 | 6 | -9.77 |
| Gefitinib | Dock to 3UE4 | Type I | - | 0 | 6 | -8.84 |
| Imatinib | 1OPJ | Type II | 1.75 | 5 | 16 | -9.19 |
| Nilotinib | 3CS9 | Type II | 2.21 | 4 | 17 | -10.74 |
| Ponatinib | 3OXZ | Type II | 2.20 | 0 | 21 | -11.70 |
| Total |  |  |  | 19 | 125 |  |

####

## Table S2. Performance of regressors on cross-validation and blind tests with and without hypothetical reverse mutations. The models were trained using 10-fold and leave-one-position out cross validation schemes. The best performing model is highlighted in bold, which is the final model in SUSPECT-ABL.

| **Mutations in the training set** | **Cross-validation** | **Blind test**  **Pearson (r)** | **Training**  **Pearson (r)** |
| --- | --- | --- | --- |
| Forward | 10-fold cross validation | 0.70 | 0.76 |
|  | Leave-one-position out | 0.67 | 0.75 |
| Forward and reverse | 10-fold cross validation | 0.73 | 0.80 |
|  | **Leave-one-position out** | **0.75** | **0.78** |

##

## Table S3. Selected features in the final model of regressor.

|  | **Feature** | **Description** | **Category** |
| --- | --- | --- | --- |
| 1 | ATP_Inter-Neut:Pos-5.00 | Proportion of protein-ATP interactions between neutral and positive atoms within 5 Å | Graph-Based Signature (ABL1-ATP) |
| 2 | KOSJ950100_SST | Evolutionary information relies on optimal substitution matrix | AAIndex |
| 3 | Inter-Acc:Acc-5.00 | Proportion of protein-drug interactions between acceptor atoms within 5 Å | Graph-Based Signature (ABL1-inhibitor) |
| 4 | Hydro:Neut-5.00 | Proportion of intra-residual interactions between hydrophobic and neutral atoms within 5 Å in drug bound protein | Graph-Based Signature (ABL1-inhibitor) |
| 5 | Inter-Don:Hydro-4.00 | Proportion of protein-inhibitor interactions between donor and hydrophobic atoms within 4 Å | Graph-Based Signature (ABL1-inhibitor) |
| 6 | LIG.POSIONIZABLE_COUNT | #positive ionizable atoms in the drug | Inhibitor |
| 7 | Acc:Hydro-6.00 | Proportion of intra-residual interactions between acceptor and hydrophobic atoms within 6 Å in drug bound protein | Graph-Based Signature (ABL1-inhibitor) |
| 8 | LIG.NUM_ROTATABLE_BONDS | #rotatable bonds in the drug | Inhibitor |
| 9 | ATP_Aro:Neg-7.00 | Proportion of intra-residual interactions between aromatic and negative atoms within 7 Å in ATP bound protein | Graph-Based Signature (ABL1-ATP) |
| 10 | ATP_Neut:Pos-2.00 | Proportion of intra-residual interactions between neutral and positive atoms within 2 Å in ATP bound protein | Graph-Based Signature (ABL1-ATP) |

##

## Table S4. Performance of the classifier (with the cutoff 1.36 Kcal/mol) on blind test and training sets with and without the hypothetical reverse mutations. The model was trained using 10-fold and leave-one-position out cross validation methods. Mutations having a binding affinity value of greater than 1.36 Kcal/mol were considered resistant.

| Mutations in the training set | Cross validation method | Blind test  MCC | Training  MCC |
| --- | --- | --- | --- |
| **Forward** | 10-fold cross validation | 0.63 | 0.68 |
|  | **Leave-one-position out** | **0.63** | **0.73** |
| Forward and resistant reverse | 10-fold cross validation | 0.56 | 0.62 |
|  | Leave-one-position out | 0.56 | 0.62 |

##

## Table S5. Selected features in the classifier (with the cut-off 1.36 Kcal/mol).

|  | **Feature** | **Description** | **Category** |
| --- | --- | --- | --- |
| 1 | Acc:Don-6.00 | Proportion of intra-residual interactions between acceptor and donor atoms within 6 Å in drug bound protein | Graph-Based Signature (ABL1-inhibitor) |
| 2 | Inter-Don:Neg-10.00 | Proportion of protein-drug interactions between donor and negative atoms within 10 Å | Graph-Based Signature (ABL1-inhibitor) |
| 3 | Inter-Hydro:Neut-9.00 | Proportion of protein-drug interactions between hydrophobic and neutral atoms within 9 Å | Graph-Based Signature (ABL1-inhibitor) |
| 4 | LIG.NUM_DONORS | #donors in the drug | Inhibitor |
| 5 | ATP_Aromatic | Aromatic interactions between ATP and protein | Arpeggio (ABL1-ATP) |
| 6 | d_Amide-Ring | Changes in Amide-Ring interactions between drugs and protein upon mutations | Arpeggio (ABL1-inhibitor) |
| 7 | Neut:Pos-4.00 | Proportion of intra-residual interactions between neutral and positive atoms within 4 Å in drug bound protein | Graph-Based Signature (ABL1-inhibitor) |
| 8 | LIG.TPSA | Topological polar surface area in the drug | Inhibitor |
| 9 | Dynamut2 | Protein stability prediction in Kcal/mol | Protein Stability and Dynamics |

##

## Table S6. Performance of the classifier (with the cut-off 0.95 Kcal/mol) on blind test and Training sets with and without the hypothetical reverse mutations which cause resistance. The method by Hauser *et al.*^1^ is also shown for comparison purposes. Mutations having a binding affinity value of greater than 0.95 Kcal/mol were considered resistant.

| Mutations in the training set | Methods | blind test  MCC | Training  MCC |
| --- | --- | --- | --- |
| Forward | 10-fold cross validation | 0.60 | 0.69 |
|  | Leave-one-position out | 0.67 | 0.69 |
|  | *Hauser et al*.^1^ | 0.50 | 0.14 |
| **Forward and resistant reverse** | 10-fold cross validation | 0.46 | 0.53 |
|  | **Leave-one-position out** | **0.67** | **0.75** |

#

#

# **FIGURES**


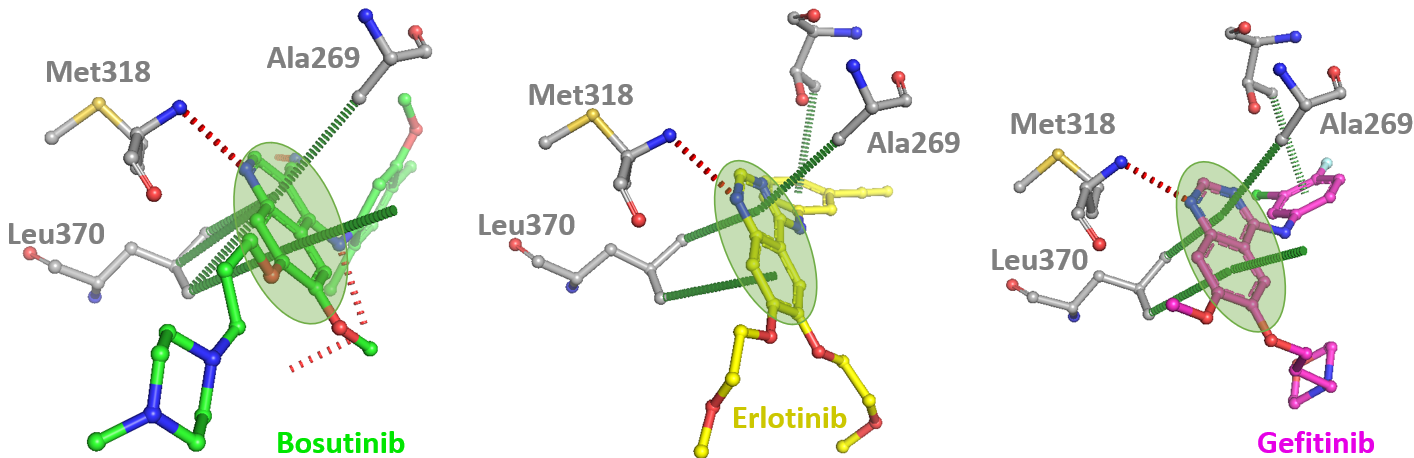


## Figure S1. Abl kinase complexed with Bosutinib, Erlotinib and Gefitinib. The Abl:Bosutinib complex (PDB ID: 3ue4, left panel), docked Abl:Erlotinib (middle panel) and Abl:Gefitinib (right panel). The chemical fragments highlighted in green oval share Carbon-π interactions (green dash line) with Leu370 and Ala269, and a hydrogen bond (red dash line) with Met318.

##
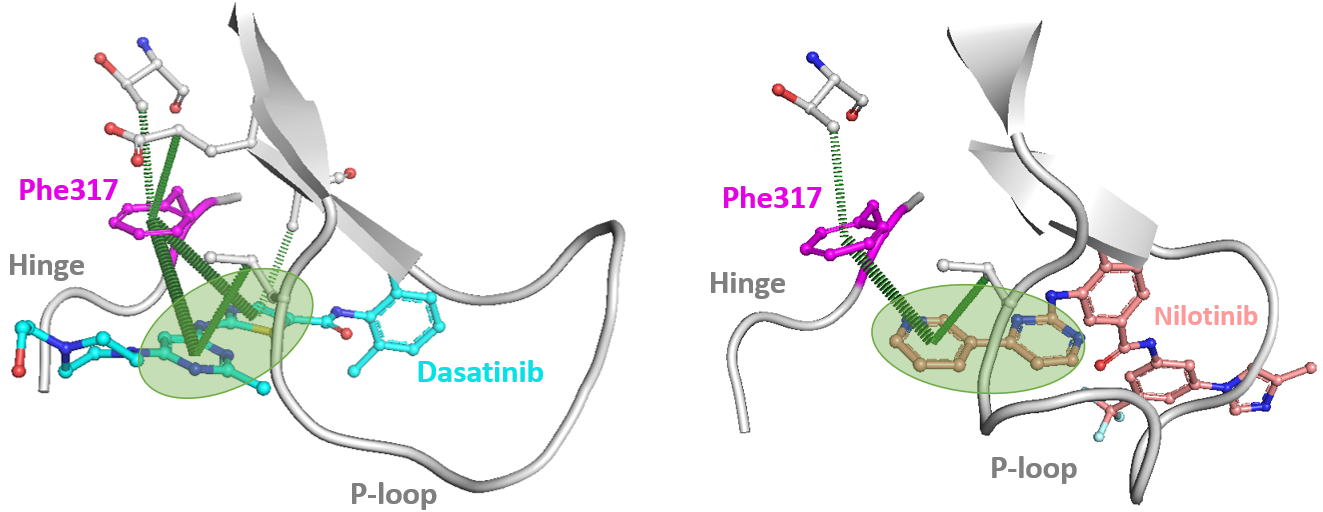


##

## Figure S2. Intermolecular interactions with Phe317 in type I and II binding modes. Type II inhibitors (*e.g.*, Nilotinib, right panel) have less contacts with the hinge region (*e.g.*, Phe 317) than type I inhibitors (*e.g.*, Dasatinib, left panel), but their adenine analogs (highlighted in green oval) are shielded by the “kinked” P-loop, making them less accessible to solvent. The Carbon-π interactions are shown in green dash lines.

##
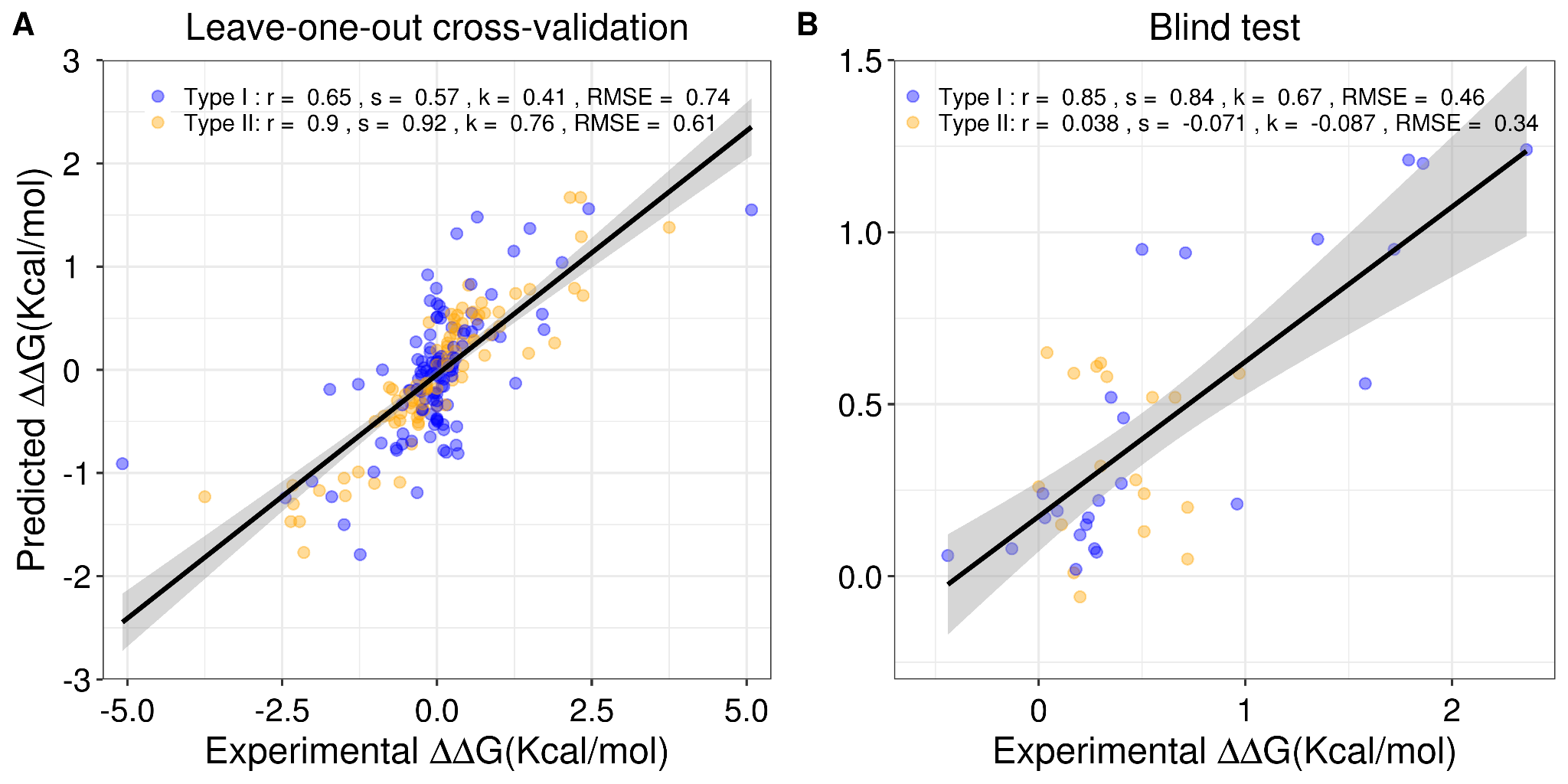


##

## Figure S3. Regression plot between actual and predicted ΔΔG colored by binding modes. The data points are the same as that of Figure 4 where type I, and II are represented in blue, and orange, respectively.


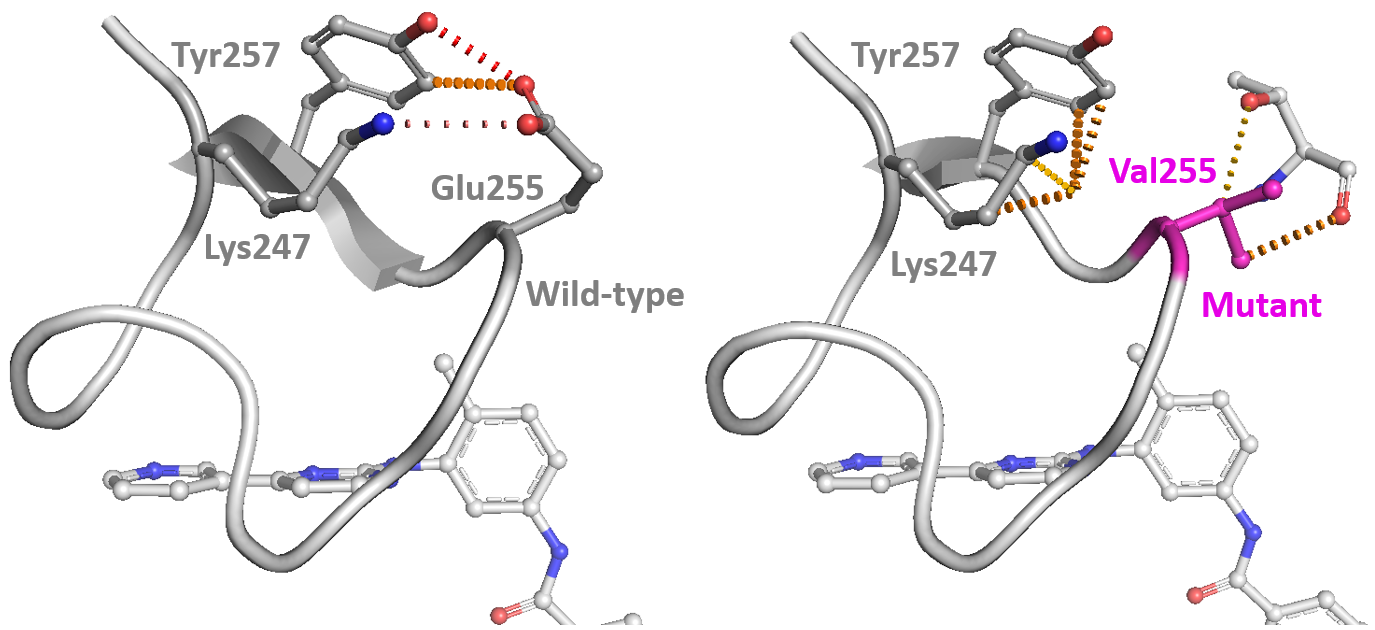


##

## ￼Figure S4. The triad interactions stabilize the P-loop. The shown structures are wild-type (left panel) and E255V mutant (right panel) ABL bound with Imatinib. The electrostatic and polar interactions between the side chains of Lys247, Glu255, and Tyr 257 are required for binding of type II inhibitors.

##

##
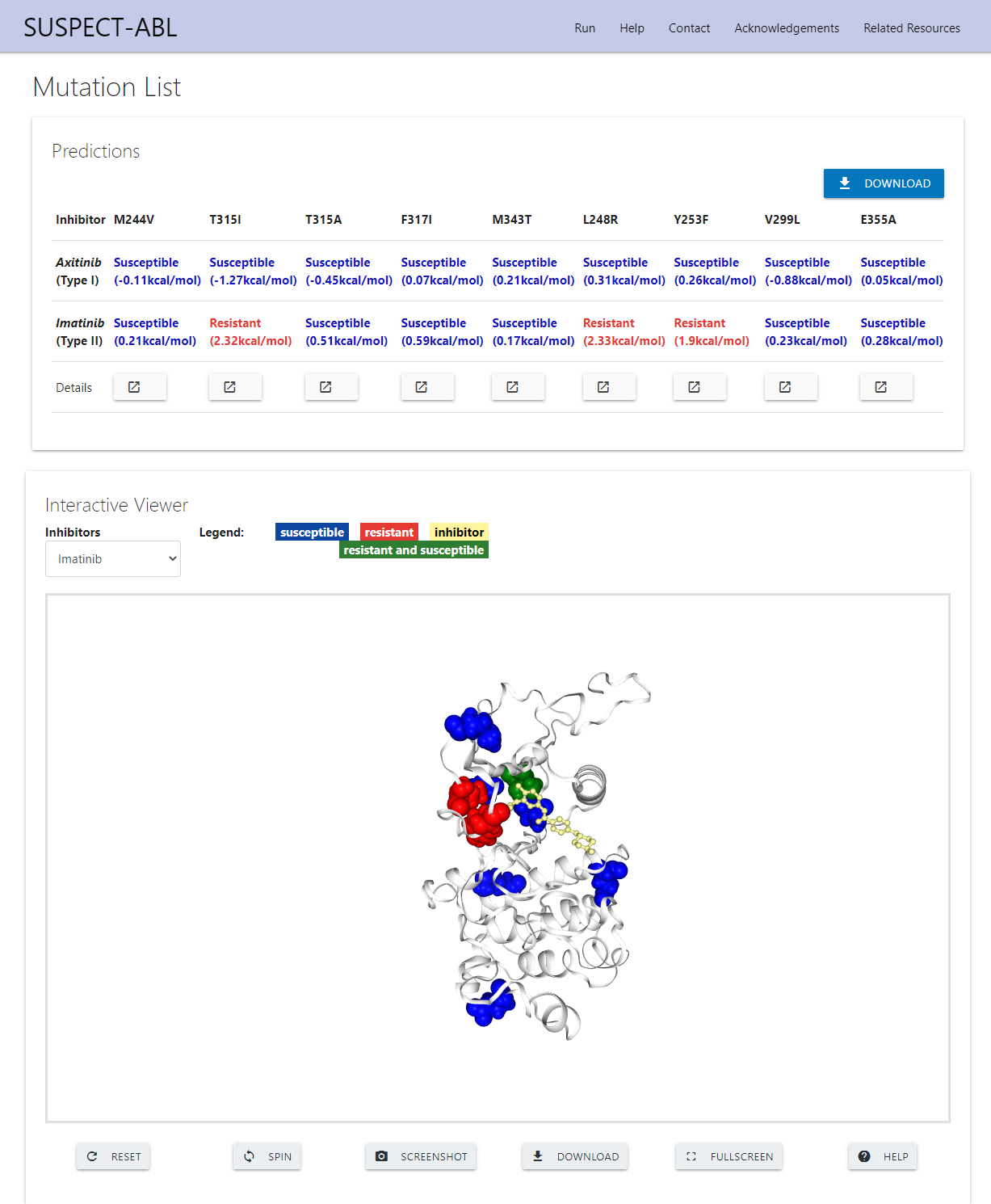


## Figure S5. SUSPECT-ABL web server result page for mutation list input. The predictions for each drug against each mutation in the input file are shown in the table. Each mutation has a detail button at the bottom, which navigates users to the result page for the corresponding single point mutation. In the interactive viewer, users can select the inhibitor of interest, and visualize the position of input mutations on the ABL structure. Mutations predicted as resistant are colored in red, the susceptible are colored in blue. In the case of different mutant residues occurring at the same position with different prediction outcomes, the site is colored in green (in this example, position 315 for imatinib).

**References**

(1) Hauser, K.; Negron, C.; Albanese, S. K.; Ray, S.; Steinbrecher, T.; Abel, R.; Chodera, J. D.; Wang, L. Predicting resistance of clinical Abl mutations to targeted kinase inhibitors using alchemical free-energy calculations. *Commun Biol* **2018,** *1*, 70.

(2) Pemovska, T.; Johnson, E.; Kontro, M.; Repasky, G. A.; Chen, J.; Wells, P.; Cronin, C. N.; McTigue, M.; Kallioniemi, O.; Porkka, K.et al. Axitinib effectively inhibits BCR-ABL1(T315I) with a distinct binding conformation. *Nature* **2015,** *519* (7541), 102.
